# Characteristics and evaluation of atherosclerotic plaques: an overview of state-of-the-art techniques

**DOI:** 10.3389/fneur.2023.1159288

**Published:** 2023-10-12

**Authors:** Zhiwei He, Jiaying Luo, Mengna Lv, Qingwen Li, Wei Ke, Xuan Niu, Zhaohui Zhang

**Affiliations:** ^1^Department of Neurology, Renmin Hospital of Wuhan University, Wuhan, China; ^2^Department of Anesthesiology, Renmin Hospital of Wuhan University, Wuhan, China

**Keywords:** atherosclerosis, biomarker, vulnerable plaque, plaque analysis, imaging method, quantitative evaluation

## Abstract

Atherosclerosis is an important cause of cerebrovascular and cardiovascular disease (CVD). Lipid infiltration, inflammation, and altered vascular stress are the critical mechanisms that cause atherosclerotic plaque formation. The hallmarks of the progression of atherosclerosis include plaque ulceration, rupture, neovascularization, and intraplaque hemorrhage, all of which are closely associated with the occurrence of CVD. Assessing the severity of atherosclerosis and plaque vulnerability is crucial for the prevention and treatment of CVD. Integrating imaging techniques for evaluating the characteristics of atherosclerotic plaques with computer simulations yields insights into plaque inflammation levels, spatial morphology, and intravascular stress distribution, resulting in a more realistic and accurate estimation of plaque state. Here, we review the characteristics and advancing techniques used to analyze intracranial and extracranial atherosclerotic plaques to provide a comprehensive understanding of atheroma.

## 1. Introduction

Cerebrovascular and cardiovascular disease (CVD) is a worldwide public health challenge and a major cause of morbidity, mortality, and economic burden. Vascular events, such as myocardial infarction and ischemic stroke, severely impair quality of life and are life-threatening. Atherosclerosis is a major contributor to the development of CVD (1). Although the incidence of cardiovascular events related to atherosclerosis can be effectively reduced by approximately 50% through lipid level control, the ongoing risk of plaque events remains a significant concern (2).

The two main characteristics of atherosclerosis are increased plasma low-density lipoprotein (LDL) levels and vascular wall inflammation (3). Atheromatous plaques are formed gradually by the deposition of lipids into the subintima of arteries, with the participation of vascular smooth muscle cells (VSMCs) and macrophages (4). Vulnerable plaques exhibit notable features such as elevated inflammation, neovascularization, intraplaque hemorrhage (IPH), a large lipid core, and a thin fibrous cap (5).

A comprehensive scientific evaluation of atherosclerosis is crucial for preventing CVD. The progression of atherosclerosis is accompanied by plaque formation and vascular remodeling. The analysis of various types of plaque characteristics and vascular hemodynamics provides a basis for identifying at-risk plaques and guiding interventions. Conventional imaging methods, including computed tomography (CT), magnetic resonance imaging (MRI), and ultrasonography, are currently used to determine various plaque characteristics from different perspectives. However, these methods only provide a cross-sectional snapshot of the current state, disregarding essential properties known to be critical determinants of future risk. To address these challenges, emerging invasive and noninvasive imaging techniques, including intravascular ultrasound (IVUS), optical coherence tomography (OCT), and near-infrared spectroscopy (NIRS), are gaining prominence. Each of these methods provides unique insights into the various aspects of plaque composition. Consequently, our objective was to comprehensively review the physical characteristics of plaques and the methods employed for their assessment with the aim of enhancing our understanding of their role in atherosclerosis and CVD.

## 2. Characteristics of atheroma

### 2.1. Pathophysiology of atherosclerosis

Atherosclerosis is a chronic inflammatory disease in which changes in wall shear stress (WSS) and LDL infiltration are pivotal factors. WSS refers to the tangential force exerted by blood on the vessel wall as it flows parallel to the direction of blood flow (6). Under physiological conditions, WSS promotes endothelial cell (EC) growth and inhibits the expression of inflammatory factors and inflammation-related pathways (7, 8). The accumulation of lipids and foam cells in the subintima disrupts the fluidity of the vessel wall, resulting in turbulent flow and subsequent alterations in WSS (9). These alterations in WSS have detrimental effects on ECs, including impaired function and compromised integrity of the arterial intima (10, 11).

In the initial phases of subintimal lipid deposition and accumulation of atheromatous substances, compensatory vascular mechanisms prevent luminal narrowing. However, when the growth of atherosclerotic plaques exceeds the compensatory capacity of the vessels, it leads to alterations in hemodynamics within the plaque and vascular structure (12). Protrusion of the plaque into blood vessels results in narrowing of the lumen diameter, disturbing blood flow downstream of the plaque. Consequently, an area characterized by a low WSS and high oscillatory vascular stress is established, promoting the persistent growth of atherosclerotic plaques (13, 14). Conversely, regions with higher WSS may exhibit a correlation with plaque regression. Lan et al. demonstrated a significant association between the total area of the high WSS region encompassing the stenotic lesion and the area of the high WSS region proximal to the lesion with regression of symptomatic intracranial atherosclerotic plaques (15).

**Figure 1:**
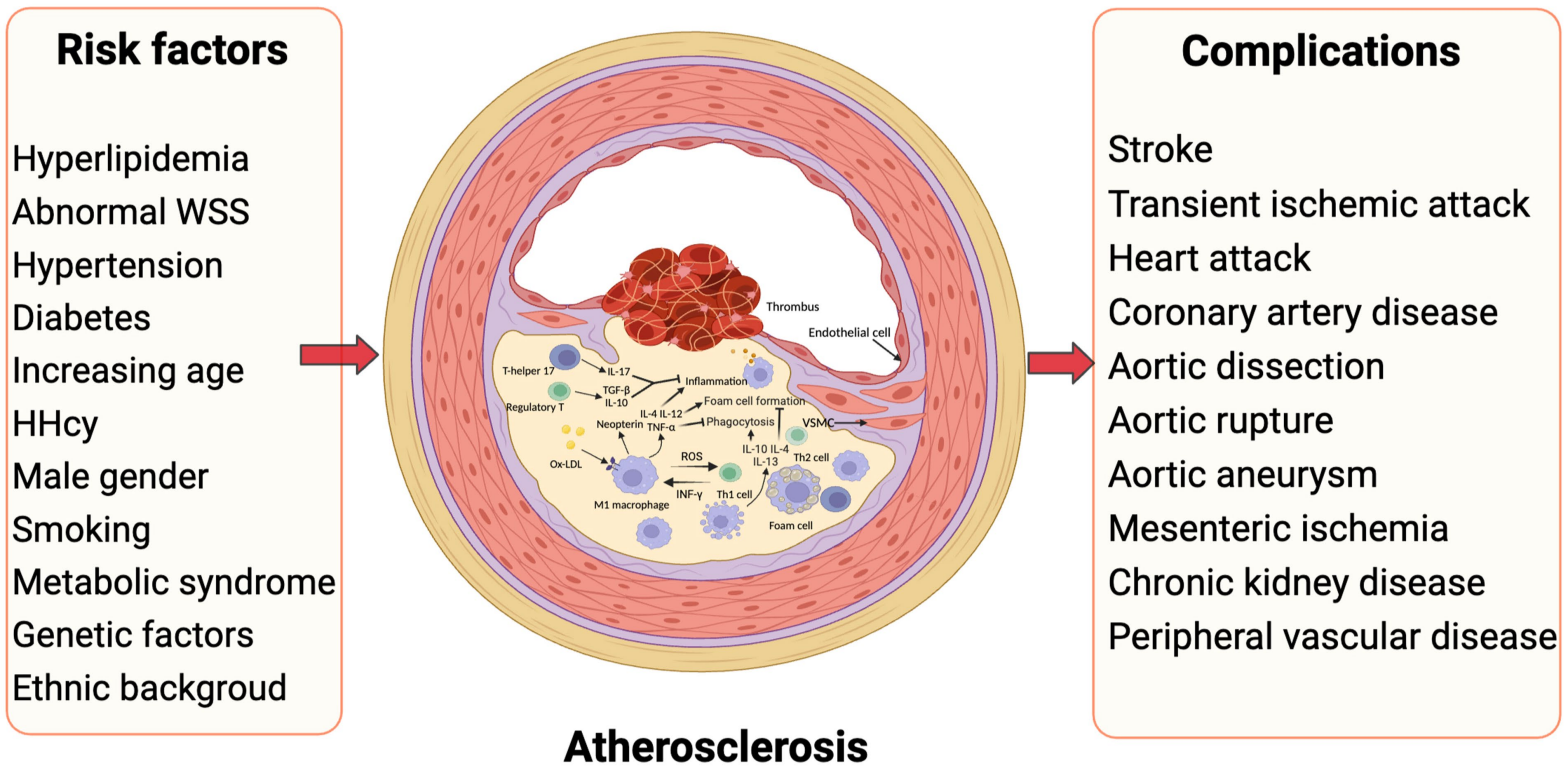
Inflammatory atherosclerosis, risk factors and subsequent complications. Traditional risk factors such as dyslipidemia, hyperglycemia, and hypertension play a crucial role in the development of atherosclerosis. Moreover, alterations in vascular blood flow, particularly abnormal WSS, contribute significantly to this pathological process. The presence of local inflammation in blood vessels is observed consistently throughout the progression of atherosclerosis, involving multiple cytokines and cell types. Ultimately, the rupture of atheromatous plaque in various arteries leads to the manifestation of diverse atherovascular diseases. HHcy, hyperhomocysteinemia; WSS, wall shear stress (Created with BioRender.com).

Within the subintima, LDL infiltrates and undergoes gradual oxidation as it lacks sufficient protection from antioxidants (16). Oxidized low-density lipoprotein (ox-LDL), formed by LDL in the presence of oxides such as reactive oxygen species, is a strong ligand for macrophage scavenger receptors (cluster of differentiation (CD) 36, SR-AI/II, and SR-BI), contributing to their uptake into macrophages (17). Furthermore, ox-LDL promotes smooth muscle cell migration from the tunica media (via platelet-driven growth factor and basic fibroblast growth factor) to sites of lipid deposition and abnormal proliferation (via insulin-like growth factor 1 and epidermal growth factor), which involves the secretion of extracellular matrix proteins (17, 18). Macrophage-like VSMCs also play a crucial role in facilitating the phagocytic clearance of ox-LDL and contribute to the formation of foam cell-like VSMCs that strongly associate with the lipid core (19). This combination of factors results in the progressive formation and development of a necrotic core of the plaque (Figure 1).

The dynamic equilibrium between plaque regression and progression is influenced by various biological processes, including inflammation, cell death, extracellular matrix degradation, and connective tissue repair responses. Plaque regression usually results from reduced plaque lipid content, LDL levels, and inflammatory status (20, 21). When lipid-lowering therapy reduced LDL levels to less than 70 mg/dL, investigators observed a reduction in the plaque lipid core area and significant plaque regression (22, 23). The classic lipid-lower statins not only inhibit LDL synthesis but may also promote the polarization of M2-like macrophages (24). M2-like macrophages accumulate at the sites of injury and release mediators that suppress inflammation in plaques, clear apoptotic cells, and promote tissue repair (25). It’s worth noting that Faisel et al. discovered that plaque regression in the carotid artery was commonly observed in larger plaques with fewer fibrotic characteristics, which indicate that plaque remission may not necessarily correspond to reduced cardiovascular risk (26).

### 2.2. Features of culprit plaques

The risk of different plaque events progressively increases as lipids and atheromatous material accumulate in the subintimal region. Disease progression is significantly influenced by plaque erosion, rupture, thrombosis, neovascularization, and IPH.

Neovascularization is characterized by immature blood vessels with sparse or even absent VSMCs and a lack of tight junctions between ECs, resulting in leakage of blood components from the vessels (27, 28). Hypoxic conditions within the plaque stimulate the release of vascular endothelial growth factor, promoting neovascularization, elevating microvessel density, and disrupting vascular organization (29). Moreover, in advanced atherosclerotic lesions, matrix metalloproteinase (MMP)-9 and plasma myeloperoxidase play a crucial role in promoting oxidation and inflammation, which consequently result in the degradation of the fibrous cap and compromises the stability of plaques. These factors ultimately facilitate the formation of IPH and infiltration of blood cells, which further promote plaque rupture. Zhao et al. reported that IPH was independently associated with a significant increase in lipid core volume (percentage difference in relative lipid core volume change: 50.3%/year, 95% CI: 19.4, 89.2, *p* < 0.001) and plaques with IPH had a greater decrease in lumen area than plaques without IPH (mean: −0.4 ± 0.9 versus 0.3 ± 1.4 mm^2^/year, *p* = 0.033) (30). This suggests that plaques with IPH undergo a transition into vulnerable plaques. After correcting for traditional risk factors, plaque load, and volume of the lipid-rich necrotic core, IPH volume remained significantly associated with the risk of acute ischemic stroke on the ipsilateral side (31). Bos et al. also observed a higher risk of stroke in patients with carotid IPH (32).

**Figure 2:**
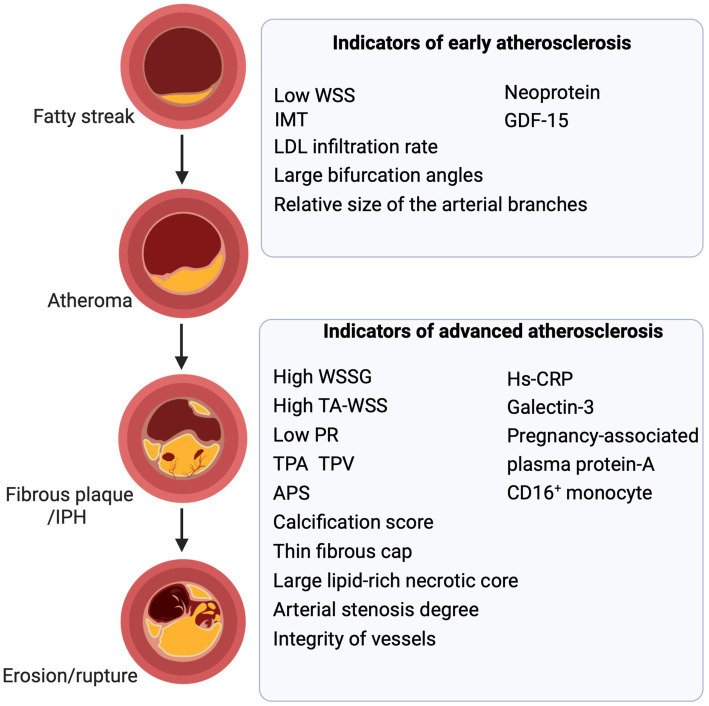
Biomarkers and parameters of atherosclerosis. Biomarkers, imaging parameters, biomechanical parameters, and anatomical parameters act as valuable indicators at distinct stages of atherosclerosis. Combining them to assess atheromatous plaques and vessels is fundamental and aids in predicting and guiding treatment. WSS, wall shear stress; IMT, intima-media thickening; LDL, low-density lipoprotein; GDF-15, growth differentiation factor 15; IPH, intraplaque hemorrhage; TPA, total plaque area; TPV, total plaque volume; WSSG, wall shear stress gradient; TA-WSS, time-averaged WSS; APS, the axial plaque stress; PR, pressure ratio; MMP, matrix metalloproteinase (Created with BioRender.com).

Plaque erosion, although milder than plaque rupture, is also associated with plaque stability. It disrupts the structural integrity of the fibrous cap of the plaque, leading to the exposure of its contents to the bloodstream. Consequently, plaque erosion often coincides with thrombotic-like plaque rupture. Upon analyzing OCT images of 115 patients with acute coronary syndrome, Kato et al. found that plaque erosion may already be present in early atherosclerosis (33). Patients with plaque erosion showed less positive vascular remodeling and neovascularization, indicating that plaque erosion is an early warning sign of plaque rupture [(34); Figure 2, left panel].

Disruption of calcium and phosphorus homeostasis occurs in foam and smooth muscle cells within microdomains after apoptosis. In addition, calcification-associated extracellular matrix vesicles aggregate to form foci of microcalcification (35). Moreover, differentiation of VSMCs into calcified cells is intricately linked to inflammatory and oxidative responses. These observations suggest that plaque calcification is a complex outcome arising from multifactorial interactions. The universal consensus is that the degree of calcification reflects the risk of developing atherosclerotic plaques. Dense calcification is generally indicative of stable plaques with a lower risk of adverse vascular events (36). Conversely, multiple small lesions featuring low-density calcification, particularly in proximity to the lipid pool and fibrous cap, signify fewer stable lesions and heightened risk of vascular events. Significant stenosis is observed in advanced atherosclerotic vessels and has been used as a predictor of myocardial infarction (37).

### 2.3. Distribution of intracranial and extracranial plaques

**Figure 3:**
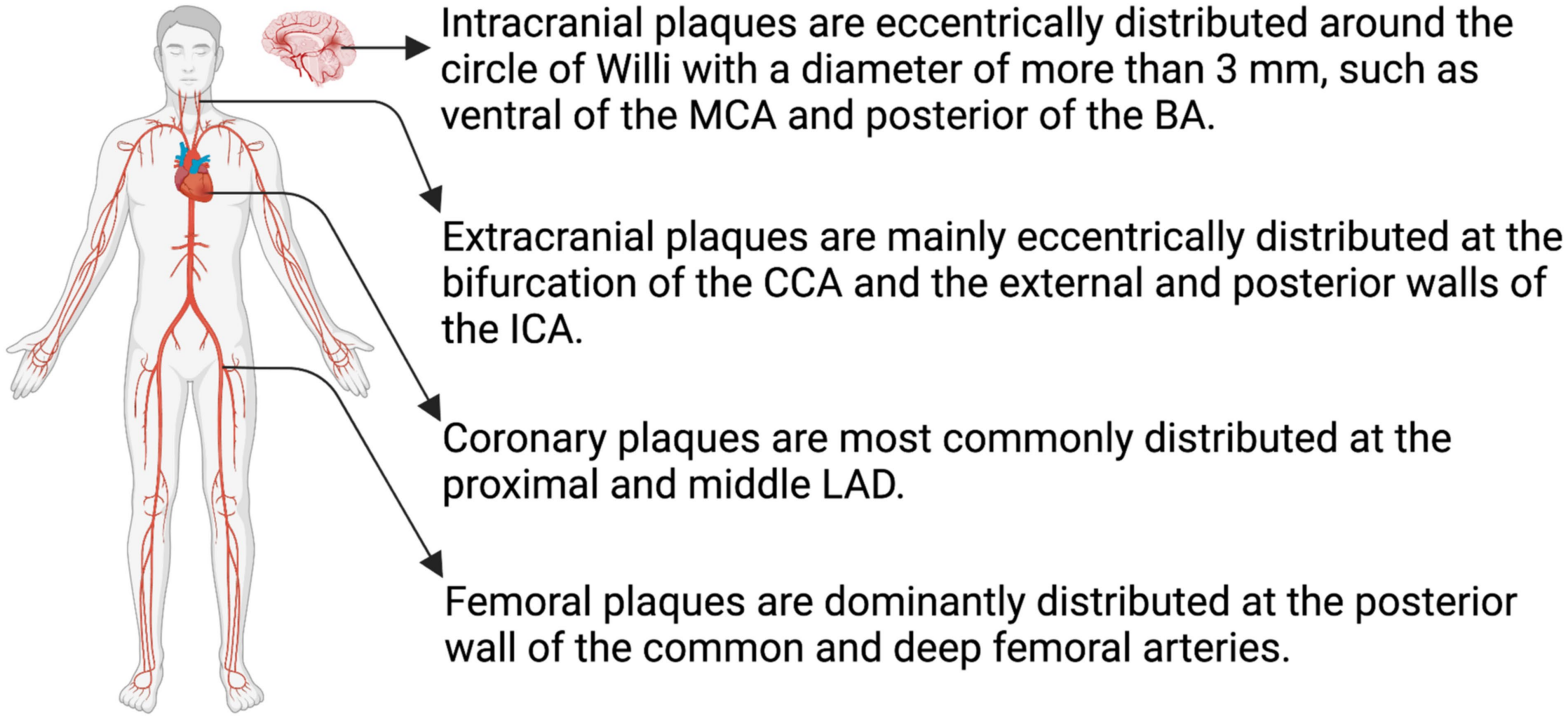
Distribution of atheromatous plaques. The distribution of atheromatous plaques varies across different arterial systems, leading to distinct characteristics in their evolution and outcomes. MCA, middle cerebral artery; BA, basilar artery; CCA, common carotid artery; ICA, internal carotid artery; LAD, left anterior descending coronary artery (Created with BioRender.com).

Atherosclerotic risk factors have a broad effect on the arterial system (38). However, plaque formation in the major large vessels is specifically influenced by factors such as hemodynamics (39), morphology (40), and variation in biochemical parameters (41). Atherosclerotic plaques mainly occur in large-and medium-sized arteries, including the carotid, coronary, femoral, and the circle of Willis arteries. Intracranial atherosclerotic plaques are eccentrically distributed in the basilar, anterior, middle, and posterior cerebral arteries and their branches, all of which have a diameter > 3 mm (42). In an observational study, Xu et al. divided the sagittal location of the middle cerebral artery (MCA) into four quadrants based on high-resolution MRI (HR-MRI): superior, inferior, dorsal, and ventral, and found that MCA plaques were more frequently located in the walls of the ventral and inferior sections (43). Sun et al. further discovered that MCA plaques were most common in the proximal M1 segment, whereas MCA plaques causing infarction were mainly distributed in the ventral and superior wall (44). Their investigation also revealed the prevalence of plaques within the distal segment of the basilar artery (BA) (44). In a subsequent analysis involving 86 patients with plaques in the BA, Zheng et al. categorized all cross-sections exhibiting eccentric plaques based on their orientation in the anterior, posterior, or lateral (left or right) center of the vessel. The results of this analysis revealed a higher likelihood of plaque distribution in the posterior wall among patients with pontine infarction [(45); Figure 3]. Patients with intracranial atherosclerosis (ICAS) have more severe strokes and longer hospitalization than those without (46).

Interestingly, ICAS exhibits a higher prevalence in the Eastern population and manifests at an earlier age than in the Western population. This difference may be partly associated with the metabolic syndrome (47). Metabolic syndrome is a chronic noninfectious syndrome characterized by a cluster of vascular risk factors, including insulin resistance, hypertension, abdominal obesity, impaired glucose metabolism, and dyslipidemia (48). Hypertension is more prevalent among Asian populations. Several gene polymorphisms associated with high salt sensitivity have been implicated as causative factors, including genes encoding α-adducin, angiotensinogen, and aldosterone synthase (49, 50). Lifestyle and diet also contribute to these differences. In Asian populations, heightened alcohol consumption and smoking are contributory factors that accelerate the progression of atherosclerosis. Excessive alcohol consumption in individuals with aldehyde dehydrogenase deficiency is associated with elevated blood pressure (49). Conversely, Western diets are closely associated with hypercholesterolemia, which is more strongly correlated with extracranial atherosclerosis than with intracranial atherosclerosis (50). Furthermore, the distribution of body fat and adiponectin levels in Eastern populations differs from those observed in Western countries (47, 51). These factors may be associated with the higher incidence of ICAS in Asian populations.

Circumferential extracranial atherosclerotic plaques rarely form in the carotid artery or its branches. Instead, these plaques tend to exhibit an eccentric distribution, primarily located at the carotid bifurcation and the external and posterior walls of the internal carotid artery (1). In coronary arteries, plaques predominantly develop in the proximal and mid left anterior descending artery, followed by proximal right coronary artery. Additionally, the proximal anterior descending artery exhibits higher plaque calcification compared to the other arteries (52, 53). Femoral artery plaques are primarily localized in the posterior wall of the common and deep femoral arteries (Figure 3). These plaques display lower levels of lipid deposition and inflammation and demonstrate a higher tendency for osteogenesis (54).

Atherosclerotic plaques may be distributed across multiple vessels. In a cross-sectional study of 3,067 adults (aged between 50 and 75 years) in southeastern China, Pan et al. observed that atherosclerotic plaques were predominantly distributed in the aortic and iliac arteries. The presence of plaque in either the aortic or iliofemoral arteries indicated an 85.3% likelihood of the plaque being present in other vascular territories (55). A similar trend was identified by Lambert et al. in people with a low to moderate risk of CVD, in which they divided the arterial tree into 31 segments and analyzed the narrowest part of each segment. They found that 27% of the participants had atherosclerosis in multiple vessels and that the atherosclerosisis relatively evenly distributed throughout the cardiovascular system, such as the abdominal aorta, iliac arteries, subclavian arteries, and femoral arteries (56).

## 3. State-of-the-art techniques

### 3.1. Advanced imaging technology

Radiographic risk factors for atherosclerosis include arterial occlusion, the degree of stenosis, and thrombotic lesions. To effectively assess the risk of CVD, researchers have persistently explored innovative examination techniques that provide comprehensive insights into plaque characteristics and vascular hemodynamics (Table 1).

**Table 1:** Imaging techniques for plaque features.

Imaging		Application
US	IVUS	Plaque location, size, and morphology (57)
	VH-IVUS	Better reflection of plaque necrotic core, thickness/rupture of fibrous cap (58)
	CEUS	IPH and neoangiogenesis: intraplaque enhancement or spot enhancement (59)
	PWI	Distinguishing between calcified and lipid plaques, predicting plaque stiffness (60)
CT		Plaque ulceration, rupture, size, distribution, calcification, vascular remodeling; incapability to accurately determine IPH, status of fibrous cap, neoangiogenesis, inflammation (61, 62)
	SPCCT	Quantifying fibrous cap thickness, area, and lipid-rich necrotic core area (63)
MRI		IPH: High signal in T_1_W, MP-RAGE, 3D TOF, 2D-FSE Calcification: low signal in TOF, T_1_W, PD, T_2_W LNRC: Unenhanced area of plaque in CE-T_1_W Fibrous cap: Thin hypointense band between LRNC and vessel lumen (61, 64)
PET	^18^F-FDG	Inflammation (65)
	^11^C-PK11195	Activated macrophage (66)
	^18^F-FOL	Activated macrophage (67)
	^68^Ga Pentixafor	Inflammation, endothelial progenitor recruitment (68)
	^18^F-NAF	Microcalcification (69)
	^18^F-Fluciclatide	Neoangiogenesis (70)
	^68^Ga-FAPI-04	Status of plaque fibrous caps (71)
OCT		Plaque calcification; thickness of fibrous cap; IV-OCT enhances identification of plaque lipid core, but complete imaging of large lipid core is difficult (72, 73).
NIRS		Quantitative assessment of plaque lipid composition (57)
NIRF		Plaque inflammation, oxidative stress, microcalcification (74)

US, ultrasound; IVUS, intravascular ultrasound; VH-IVUS, virtual histology intravascular ultrasound; CEUS, contrast-enhanced ultrasound; PWI, pulse wave imaging; CT, computed tomography; SPCCT, spectral photon-counting CT; MRI, magnetic resonance imaging; PET, positron emission tomography; OCT, optical coherence tomography; NIRS, near infrared spectroscopy; NIRF, near-infrared fluorescence; MP-RAGE, magnetization prepared rapid gradient echo; 3D TOF, 3D time-of-flight; PD, proton density; ^18^F-FDG, 2-deoxy-2-[^18^F]fluoro-D-glucose; ^18^F-FOL, fluoride-18-labeled 1,4,7-triazacyclononane-1,4,7-triacetic acid conjugated folate; ^68^Ga-FAPI-04, gallium-6837 conjugated quinoline-based FAP inhibitor; LRNC, lipid-rich necrotic core; IPH, intraplaque hemorrhage.

#### 3.1.1. Ultrasound

Ultrasonographic imaging is widely recognized as one of the most frequently used and cost-effective methods for assessing atherosclerotic plaques. However, owing to the principle of sound reflection, its sensitivity and specificity are low, which has the potential to underestimate the extent of arterial stenosis. The observed results are also influenced by the angle of the ultrasonic probe and the subjective perception of the examiner (75). Researchers have conducted IVUS and contrast-enhanced ultrasound examination to address these challenges. Placement of an IVUS probe in the vessel allows for better visualization of the vessel dimensions and plaque morphology, thus assisting with interventional treatment. Furthermore, IVUS improves the assessment of plaque characteristics such as shape, size, and location, and enables real-time observation of plaque composition and texture (57). With improvements in technology, IVUS has been further developed with the emergence of a more complete technique, virtual histology-IVUS. This reflects the fibrous component of the plaque well; however, the assessment of the necrotic component, fibrous cap thickness, and signs of rupture is still not satisfactory (58). A meta-analysis by Mishra et al. revealed that IVUS and its extended virtual histology-IVUS, are superior to CT/MRI in carotid interventions (76).

Contrast-enhanced ultrasound is a technique that involves injection of microbubbles during an ultrasonic examination to enhance the reflective signal, allowing accurate assessment of the lumen and neovascularity within carotid plaques (57). Compared to conventional contrast agents, microbubbles eliminate the limitations of radiation exposure and nephrotoxicity (77). Cui et al. observed a high incidence of neovascularization within plaques in patients with mild to moderate stenosis, as detected using contrast-enhanced ultrasonography. This observation is considered an independent predictor of future vascular events in patients with recent ischemic stroke (78).

Pulse-wave imaging (PWI) is an ultrasound-based method that demonstrates systematic variations in arterial properties throughout the cardiac cycle to assess arterial rigidity and flexibility. In an *in vitro* simulation test, PWI exhibited excellent discriminatory capability for distinguishing between soft, medium, and hard plaque materials (60). Li et al. also showed that PWI effectively differentiates between calcified and lipid plaque composition, enabling the prediction of atherosclerotic plaque stiffness (79).

#### 3.1.2. HR-MRI

HR-MRI holds significant utility in the scrutiny and assessment of vascular atherosclerotic plaques, with a particular emphasis on intracranial manifestations. HR-MRI for vessel wall visualization commonly encompasses T_1_−/T_2_-weighted imaging, proton-density imaging, or contrast-enhanced T_1_-weighted imaging with turbo/fast spin-echo sequences or black-blood techniques (80). The lipid core is identifiable as areas exhibiting isosignal intensity on T_1_-weighted images and as regions with low to isosignal intensity on T_2_-weighted images. Conversely, the fibrous component appears isointense on both T_1_- and T_2_-weighted images. Calcifications are characterized by dark signal intensity on both T_1_- and T_2_-weighted images, although their sensitivity and specificity are notably inferior to those of CT scans (80, 81). Initial IPH is discernible through high signal intensity on T_1_-weighted images and time-of-flight (TOF) imaging. In evaluating plaque inflammation, histological analyses of animal models have revealed a proportional relationship between the extent of macrophage infiltration in the vessel wall and the degree of plaque enhancement (82).

It’s worth noting that while these characteristics are readily discernible within the carotid system, the differentiation becomes notably more challenging within the narrower confines of intracranial arteries. Additionally, in the context of carotid plaques, researchers observed a preferential enhancement of the fibrous cap through gadolinium-based contrast agent (83). In evaluating intracranial vascular atherosclerosis, HR-MRI emerges as a precise tool for quantifying intracranial atherosclerotic stenosis, particularly in cases of moderate to severe stenosis. This accuracy stems from its direct visualization of both the vessel lumen and stenotic occlusive plaques, aligning well with findings from digital subtraction angiography (84). HR-MRI exhibits superior precision compared to magnetic resonance angiography when appraising BA stenosis and demonstrates efficacy in guiding endovascular interventions. Moreover, the application of contrast-enhanced HR-MRI proves adept at distinguishing between symptomatic and asymptomatic atherosclerotic plaques within the BA, surpassing conventional imaging variables and clinical risk factors in accuracy (85).

#### 3.1.3. Positron emission tomography

Positron emission tomography (PET) is a non-invasive nuclear imaging technique that utilizes tracers to evaluate various biological processes related to atherosclerosis, including intraplaque inflammation, microcalcification, and angiogenesis (65). High-risk plaques exhibit distinctive metabolic characteristics such as elevated glycolysis, augmented utilization of amino acids, and reduced fatty acid oxidation. Several tracers have been developed to identify the differences in the molecular expression and metabolic profiles of cells.

The uptake of fluorine-18-fluorodeoxyglucose reflects the glucose metabolism status of tissues, which correlates with the levels of plaque inflammation. However, this method may result in false positives owing to non-specific uptake. Specifically, ^11^C-PK11195, ^18^F-FOL, and ^68^Ga Pentixa have been used to detect mononuclear macrophages (66–68), providing a more accurate assessment of plaque inflammation levels. ^18^F-NAF is a tracer designed to detect active microcalcification within atherosclerotic plaques, thus facilitating the identification of vulnerable plaques (69). The function of ^18^F-Fluciclatide is intricately associated with angiogenesis through the quantification of α_v_β_3_ integrin expression. It serves as a marker for predicting plaque neovascularization and IPH (70). ^68^Ga-FAPI-04 is capable of recognizing intraplaque fibroblasts and is currently under investigation for its potential application in assessing the condition of fibrous caps (71). The evaluation of new tracers for predicting CVD necessitates large-scale randomized clinical trials; however, radiation exposure and high costs may hinder their widespread adoption and application.

#### 3.1.4. Optical coherence tomography

In atherosclerosis, OCT is employed to assess plaque calcification, neovascularization, fibrous cap thickness, and the interface between the plaque and vessel wall (86). OCT provides cross-sectional images of the arterial wall with a superior resolution of 1–15 μm, compared to a spatial resolution of approximately 100 μm for IVUS (72). Lipid plaques are characterized by the presence of diffuse signal-poor areas (lipid pools) and signal-rich bands (fibrous caps) with high signal attenuation on OCT (86). Nevertheless, capturing a comprehensive image of the lipid pool is often difficult because of limited penetration depth (72). Recent studies have attempted to employ OCT to assess cholesterol distribution within plaques (high-intensity, thin-linear regions, usually near lipid patches), but unfortunately showed low sensitivity (87, 88). Shindo et al. assessed the morphological characteristics of carotid plaque rupture by OCT and showed that a carotid plaque cap thickness < 130 μm was the threshold for plaque rupture and that most instances of rupture were in the shoulder of the carotid plaque in 36 patients with high-grade stenosis (89). Intravascular OCT offers a more comprehensive approach that integrates time-series deep learning and achieves an impressive 89.6% accuracy in identifying plaque lipid cores, significantly enhancing identification efficiency (73). High-resolution clear imaging of the vessel wall and plaque morphology has also been applied to assist in stent implantation and assess stent status (90).

#### 3.1.5. Near infrared spectroscopy and near-infrared fluorescence

Near-infrared fluorescence utilizes imaging based on the differences in the absorption and reflection of near-infrared light (wavelengths ranging from 800 to 2,500 nm) in different tissues. NIRS provides a quantitative evaluation of the lipid composition of plaques, thus compensating for the shortcomings of OCT. NIRS-based measurement of the maximum lipid core burden index within a 4 mm segment offers a quantitative estimation of lipid core size, exhibiting a strong positive linear correlation with the pathological evaluation of carotid artery stenosis (91, 92). Moreover, meta-analysis findings support the efficacy of this metric in quantifying and identifying individuals at high risk of plaque rupture and future major cardiovascular events (93). However, this technique does not assess the fibrous cap thickness, plaque burden, and vascular wall of the plaques. In view of this, researchers have integrated it with other techniques such as OCT and IVUS to comprehensively obtain plaque characteristics (57). Near-infrared fluorescence (NIRF) is an emerging technique for intravascular imaging. Utilizing fluorescent conjugates that selectively label particular cell types, near-infrared excitation light (650–900 nanometers) stimulates fluorescent agents, whereby the signal produced by the attenuation of fluorescent agents is acquired for imaging. Visualization of the inflammatory level, oxidative stress, endothelial permeability, and microcalcification of plaques can be achieved using different fluorescent labels (74). Yudai et al. validated the role of the Peptide-ICG2 (a fluorescent tracer targeting macrophages) in detecting atherosclerotic plaques vulnerable to embolism in mice (94). Researchers have also discovered many fluorescent dyes, including LO1-750, FTP11-Cy7, and osteoSense750 (74, 95). Currently, indocyanine green is the only fluorescent dye approved by the US Food and Drug Administration for human use, and its application in detecting atherosclerotic plaques is anticipated (74).

To compensate for the limitations of single imaging modalities, multimodal imaging integrates multiple techniques to achieve a more comprehensive evaluation of plaque morphology. Novel imaging modalities, such as IVUS-NIRF, OCT-NIRS, OCT-NIRF, IVUS-OCT, IVOCT-NIRF, and IVOCT-NIRS, are currently undergoing preclinical evaluation. We expect further progress in these studies to assess vulnerable carotid plaques more accurately.

### 3.2. 3D reconstruction and quantitative evaluation of plaque morphology

Although the current diagnostic standards and guidelines for coronary artery atherosclerosis are mainly related to geometric parameters derived from two-dimensional images of the coronary arteries, the characteristics of 3D reconstructed plaques have also received increasing attention.

Based on various types of imaging examination, researchers have used computer simulation technology to determine the characteristics of plaques *in vitro*. Through 3D reconstruction of plaques using carotid ultrasound, Spence et al. quantified the area and volume of plaques and described the relationship between plaque texture algorithms and plaque calcification (96). Another study by the same team confirmed the accuracy of plaque volume measurement (97). The scope of interest within 2D ultrasound is constrained by the movement of the ultrasound probe, thereby posing challenges in achieving clear visualization of the target site. Measurements pertaining to the volume of tissue or lesions are inclined to be less precise, often contingent upon the proficiency of the operator. While 2D ultrasound generates a Doppler report encompassing various hemodynamic measurements, it falls short of delivering a comprehensive depiction of the arterial anatomy along with precise localization details. Conversely, 3D ultrasound offers an intuitive and efficient means to visualize tissues or plaques in three dimensions, alleviating operator fatigue and enhancing diagnostic precision (98, 99).

Currently, 3D imaging of atherosclerotic plaques provides a comprehensive depiction of the arterial lumen, external vessel wall, and calcified plaque (volume, surface area, and maximum length). This approach significantly minimizes the need for invasive methods of examination and their associated impact on patients (100). Guo et al. used 3D HR-MRI to investigate vascular remodeling and plaque morphology in patients with severe vertebrobasilar stenosis (101). In addition, another study compared an HR-MRI-based 3D carotid plaque radiomics model constructed using the radiomic features of 3D T1-SPACE sequences with their contrast-enhanced counterparts using a conventional imaging model. The results revealed that an HR-MRI-based 3D carotid radiomics model exhibited improved accuracy in detecting vulnerable carotid plaques (102). Furthermore, Becher et al. employed Adipo-Clear and immunolabeling in conjunction with light-sheet microscopy to conduct a 3-D reconstruction of mouse cerebral arteries. In contrast to traditional inspection, this approach enables the acquisition of valuable information regarding the plaque shape, total volume, cellular volume, and cell-free volume (103). In intracranial atherosclerosis, the utilization of 2D black-blood techniques for visualizing angulated lesions or intricate intracranial arteries might hinder the attainment of cross-sectional images that are perpendicular to the arterial longitudinal axis. Conversely, the implementation of through-multiplanar 3D reconstruction empowers researchers to examine intracranial plaques from various orientations, boasting a heightened spatial resolution. Moreover, this approach facilitates the capture of cross-sectional images precisely at the location of maximal luminal stenosis, as well as the proximal and distal reference sites. Notably, the 3D technique effectively circumvents tilt artifacts, a proficiency absents in 2D scans that, in turn, could lead to an overestimation of both true wall thickness and vessel area (101, 104).

OCT-based reconstruction methodology and computational fluid dynamics (CFD) simulations are utilized for the computation of local hemodynamic quantities. Migliori et al. utilized time-averaged WSS (TA-WSS) to depict the blood flow characteristics around plaques before and after stent implantation surgery. This technique provides a remarkable and reliable visual image of blood flow around the plaque before and after stenting. An increase in the lumen cross-sectional area downstream of the lesion resulted in significant recirculation of the stream. Post-percutaneous coronary intervention surgery, a smooth lumen surface and well-apposed stent struts facilitated unobstructed blood flow without recirculation (105). This suggests that the combination of 3-D reconstruction and CFD simulation provides a visually compelling assessment of the therapeutic efficacy of stent implantation surgery.

The utilization of 3D reconstruction techniques to forecast plaque progression hinges on the formulation of the model itself. The precision of this endeavor is greatly influenced by the approach to construction and the methodology of correction. Errors that arise possess the potential to reverberate significantly, exerting a profound impact on the ultimate outcomes. While the propagated error in shear stress calculations appears negligible, it assumes a substantial magnitude when it comes to several variables within the plaque growth model. These inaccuracies in prognosticating plaque development could be attributed to several factors: (1) divergences in the reconstructed vessel geometry; (2) alterations in pressure gradients between the epithelial and endothelial boundaries; and (3) disparities between the assumptions regarding the initial concentration of VSMC within the arterial wall and the physiological reality (106).

### 3.3. Computational simulation: evaluations of hemodynamic and biomechanical parameters

Integrating various examination techniques and computer simulations, researchers have developed a variety of ‘virtual reality’ techniques to detect atherosclerotic plaques and blood vessels (107).

In 3D models, researchers estimate almost any hemodynamic parameter, including WSS, flow velocity around plaques, and carotid stenosis (107–109). Owing to the complexity of plaque geometry and composition, constructing a 3D plaque model is time consuming. Some researchers have proposed a 1-D/3-D hybrid model that aims to balance computational efficiency without compromising the accuracy of hemodynamic indicators (110). It should also be noted that because of the assumed rigid structural properties of the vessel wall, the WSS obtained using 3D models was higher than the actual value (111). Subsequently, a fluid–structure interaction (FSI) model was proposed to address the above shortcomings.

Using the FSI model, researchers have simulated the effects of atherosclerosis on the vascular and blood flow states, WSS, LDL permeability, vulnerable plaques, and surrounding features (112). Pakraven et al. employed the FSI model to investigate the status of coronary artery ECs and discovered that areas prone to atherosclerosis exhibited at least one of the following three attributes: low time-averaged WSS, high WSS angles, and high longitudinal strain (113). Using the FSI model based on IVUS and OCT, Guo et al. acquired more accurate WSS, stress, and strain. The integration of plaque morphology from OCT and IVUS along with mechanical risk factors from the FSI model yielded the highest sensitivity and specificity for predicting plaque progression (114). The complementary nature of these techniques has substantially enhanced their accuracy in predicting plaque progression and assessing cardiovascular risk.

## 4. Vascular and plaque parameters

### 4.1. Multidimensional geometric parameters

#### 4.1.1. Geometric parameters of the plaque

The intima-media thickness (IMT) is frequently used to characterize the early stages of atherosclerosis. It is strongly associated with vascular events and is a valuable predictor of early atherosclerosis (115). IMT is measured as the perpendicular distance extending from the upper margin of the intimal layer to the upper boundary of the adventitial layer on the posterior vessel wall, i.e., the combined thickness of the intima and the intima-media of the vessel wall. Some consensus suggests measuring the bilateral common carotid arteries more than 5 mm from the carotid bifurcation in the vessel wall at a distance relative to the ultrasound probe. The patient should be in dorsal recumbency with the head tilted 45 degrees to the contralateral side, and measurements should be taken during cardiac diastole (116, 117). In healthy adults, there was good agreement between B-mode ultrasound and radiofrequency, with an interobserver correlation coefficient of 0.87 (118). George et al. found that carotid IMT is a predictor of clinical coronary artery disease and is associated with the serum levels of total cholesterol and LDL (119). Furthermore, it can be evaluated using relatively simple and convenient modalities, such as color Doppler and two-dimensional ultrasound (120). The American Society of Echocardiography has reached a consensus suggesting that a carotid IMT measurement of 1.5 mm or more in any segment of the carotid artery is a criterion for determining the presence of diffuse-type plaque lesions (121). The endothelial layer and subendothelial matrix (intima) comprise only 20% of the IMT, with the remaining 80% made up of smooth muscle cells (mesothelium). IMT is strongly correlated with age and hypertension. In a 2021 meta-analysis involving 119 clinical trials, investigators found that the degree of intervention effect on carotid IMT progression predicted the degree of CVD risk reduction (122). And measurements of IMT at multiple carotid system sites better predicted cardiovascular risk (123). Compared to two cardiovascular risk calculators (Omnibus Risk Score-ORS and Framingham Risk Score-FRS), carotid IMT better predicted stroke risk and there was a positive correlation between common carotid IMT and stroke events (124, 125). It is worth noting that there is some current opinion that IMT is only a weak predictor of risk and changes very little over time (126). On a comprehensive basis, IMT remains an important indicator of atherosclerosis and has the ability to predict the risk of cardiovascular events even in advanced stages of atherosclerosis. Thickening of the intima can be caused by aging and hypertension, in addition to atherosclerosis; therefore, certain researchers propose that carotid IMT should be regarded as indicative of advanced organ disease rather than solely a preclinical stage of atherosclerosis (121, 127). The relationship between IMT and atherosclerosis may be modified in the future as more comprehensive studies are implemented.

The total plaque area (TPA) is measured by tracing the perimeter of a plaque in longitudinal section, in the plane in which it is biggest. The sum of all plaque areas in the observation area is TPA. In two-dimensional ultrasound, the selection of a specific cross-sectional slice is often affected by the imaging angle and subjective human factors, rendering it less stable than IMT measurements. Total plaque volume (TPV) measurement relies on 3D ultrasound and computer technology (128). In the study conducted by Landry et al., the researchers employed two distinct approaches to assess TPV. Initially, they utilized both a predetermined inter-slice distance (ISD) and an ISD derived from plaque endpoints. In the former approach, plaques were cross-sectioned perpendicular to the longitudinal axis of the vessel in the 3D ultrasound image. Subsequently, the contiguous contour area was averaged and then multiplied by the ISD, yielding incremental volumes as per a specified equation. The accumulation of these incremental volumes yielded the measurement of the overall plaque volume. Rigorous examination was performed to confirm the adequacy of the outlined contours in encompassing the complete plaque volume. Conversely, the latter approach involved a longitudinal assessment of the plaque to pinpoint its termination point. Here, the determination of ISD involved dividing the plaque length by an integer value representing the number of slices. This subsequently facilitated the calculation of the total plaque area. Upon a comparative analysis of these two methodologies for measuring plaque volume, the researchers observed that the coefficients of variation for TPV, as determined by the two approaches, exhibited a diminishing trend with increasing plaque volume. Notably, the TPV obtained through the former technique was consistently smaller than that derived from the latter approach. This discrepancy could be attributed to limitations inherent in the former approach, particularly in accurately identifying the distal extremity of the plaque (129). The processing and retouching process of the images was further optimized by algorithms to achieve semiautomatic measurement of TPV (130). By commencing the measurement protocol with reference to the point of carotid bifurcation, researchers effectively mitigated the extent of measurement variability. The approach has been successfully implemented in the context of carotid MRI studies (129, 131).

Compared to IMT, TPA and TPV account for overall atherosclerosis and are better predictors of plaque event risk. Owing to its slow changes over time, IMT exhibits lower sensitivity in capturing disease evolution than TPA and TPV. With a growth rate of approximately 0.15 mm/yr and a minimum carotid ultrasound resolution of 0.6 mm, changes in IMT require a significant amount of time for observation. Conversely, TPA and TPV exhibited changes at rates of approximately 10 mm^2^/yr and 50–100 mm^3^/yr, respectively. This demonstrates that TPV is the most effective measure for evaluating the treatment response (plaque reduction and plaque growth) (132).

#### 4.1.2. Geometric parameters of the artery

Vascular structural variability, including bifurcation angle, relative size, and vessel integrity, is associated with the presence of atherosclerotic plaques. Larger internal carotid artery angles generally increase the frequency and area of blood recirculation along with lower WSS on the sinus wall, thereby increasing the risk of plaque formation (38, 133). In carotid artery structure simulation studies, an increased diameter of the vessel branches corresponded to a larger blood return zone. At the bifurcation point of the carotid artery, the internal carotid artery had a larger diameter than the external carotid artery. Consequently, this discrepancy in diameter led to a relatively lower WSS and a wider blood return zone. This observation may explain the higher prevalence of plaques in the internal carotid artery (134).

Observational studies have indicated a higher prevalence of incomplete Circle of Willis configurations than intact types among patients exhibiting atherosclerotic plaques in the MCA M1 segment, especially the incomplete posterior circle of Willis, which accounted for 83.9% of cases (135). On 3D-TOF-MRA images, vertebro-basilar artery geometry was qualitatively classified into four basic geometric configurations: walking, tuning fork, lambda, and no confluence. Zheng et al. observed the highest occurrence of plaques in the walking configuration of the vertebro-basilar artery. Furthermore, they discovered that patients with BA plaques in the lambda configuration exhibited significantly greater disparities in diameter between the left and right vertebral arteries (45). Arterial stent shape changes also cause local hemodynamic differences in the arteries, which may be associated with stent restenosis and CVD. Liu et al. simulated three variants of stent shape in patients with ICAS (enlarged, internally narrowed, and externally narrowed) and found that stent geometry significantly affected WSS, with the area neighboring the stent experiencing the most pronounced effects (136).

Vertebrobasilar dolichoectasia is characterized by pronounced dilatation, extension, tortuosity, or angulation of the vertebrobasilar artery, attributed to a range of diverse factors (137). Its pathogenesis is multifactorial, encompassing atherosclerosis, hypertension, developmental anomalies, and additional elements including infection (138). Among patients with vertebrobasilar dolichoectasia who have suffered a stroke, researchers have noted an elevated frequency of plaque occurrence (139). An augmentation in both the mean diameter and bifurcation height of the BA is firmly linked to the likelihood of a stroke occurrence. Excessive bifurcation angles tend to impose pulling and twisting forces on the perforating arteries of the BA, resulting in diminished blood flow (137, 140). Meanwhile, the widening of the BA diameter leads to a noteworthy reduction in blood flow velocity, thereby fostering the development of microemboli and subsequent lipid deposition. This sequence of events ultimately contributes to the formation of atherosclerosis and subsequent stroke incidents (137, 138).

### 4.2. Morphological parameters

The attenuation values on CT corresponds to the different plaque components. Some studies suggested attenuation values not exceeding 50 Hounsfield units (HU) for lipid components, whereas others suggested values below 30 or 60 HU (141–143). Although an attenuation threshold over 130 HU is commonly used to indicate calcification on two-dimensional CT, there are discrepancies in 3D reconstruction studies, where some suggest thresholds of over 300 or 400 HU for calcification (144). In 2015, Puchner et al. suggested a threshold of >180 HU for calcified plaques (145); in contrast, in 2018, Kigka et al. indicated that attenuation values over 400 HU should be considered for calcified plaques (146). Limited by the resolution of CT and polymorphisms in plaque contents, quantitative differentiation of plaque components by attenuation values is difficult. Thus, the evaluation threshold for calcified and non-calcified plaques (mixed plaques) varies among clinical studies. According to the prevailing consensus, high-density calcification is generally defined as an HU density exceeding 350, while HU values ranging from 131 to 350 indicate fibrous plaques, and a range of 31 to 130 HU corresponds to fibrofatty plaques. Additionally, HU values ranging from-30 to 30 are associated with the presence of a necrotic core (147, 148). Notably, low-density plaques (HU < 30) have been identified as the most robust predictors of myocardial infarction in patients presenting with stable chest pain (149). Coronary artery calcium (CAC) scores obtained by CT are associated with myocardial infarction risk and are independent risk factors for cardiovascular events. Liu et al. examined 19 patients with symptomatic stenosis caused by left coronary plaques and found a positive correlation between the number and volume of coronary plaques and calcification (150). Yoon et al. also showed a positive correlation between the plaque calcification score and degree of stenosis in 66 patients (151). Notably, CAC scores have been employed in guidelines as a surrogate measure for estimating the 10-year risk of cardiovascular events and for making lipid-lowering therapy decisions. Patients with CAC scores >400 demonstrated a significantly higher likelihood of developing CVD (147, 149, 152).

The thickness of the thin fibrous cap is defined as less than 65 μm, and this criterion is most commonly used to assess plaque stability (153, 154). Owing to artifacts such as edge blur and halo effects, fibrous caps are difficult to image using CT. Recently, a study demonstrated the effectiveness of spectral photon-counting CT in quantifying fibrous cap thickness, plaque area, and lipid-rich necrotic core area, showing good consistency with histopathological measurements (attenuation values in voxels distinguish plaque regions; the thickness of the regions is measured by multiplying the number and size of voxels) (63). This new technology offers promising possibilities for precise quantitative analysis of lipid cores and fibrous caps in plaques. In a preclinical study, spectral photon-counting CT identified monocyte accumulation in the arterial wall by recognizing a tracer reflecting the progression of atherosclerosis (155). The center frequency of IVUS imaging should be higher than 70 MHz to identify thin fibrous cap, which is a challenge for current IVUS imaging systems (156). In contrast, HR-MRI demonstrates the capacity to visualize the fibrous cap effectively. Instances of fibrous cap rupture are identifiable by the presence of partially obscured and irregular surfaces within T_1_-weighted or T_2_-weighted images (157).

The ability of CT to predict IPH remains unclear. Some studies have shown no significant difference in attenuation values between plaques with and without IPH on CT (158). However, Saba et al. found a statistical correlation between IPH and low HU values (HU values <25 after contrast medium administration, indicating the presence of IPH with a sensitivity and specificity of 93.22 and 92.73%, respectively) (159). It is worth stating that the difference may be explained by the presence of a lipid-rich necrotic core (61). HR-MRI effectively addresses this gap by demonstrating excellent utility. In the context of extracranial atherosclerosis, HR-MRI’s capability to identify IPH exhibits commendable intersubject reproducibility and reliability. Notably, HR-MRI displays a robust sensitivity range (81–90%) coupled with a high specificity range (74–90%) in the discrimination of IPH, fibrous cap, and lipid cores (160). These capabilities are further bolstered when employing contrast-enhanced HR-MRI. In observational studies focusing on basilar and internal carotid artery plaques indicate that IPH manifests as a signal intensity surpassing 150% of adjacent gray matter’s signal intensity in T_1_-weighted images (80, 157).

### 4.3. Novel hemodynamic and biomechanical parameters

In the past decade, mathematical modeling and simulation have emerged as valuable noninvasive tools in the field of cardiovascular disease, aiding both basic scientific research and clinical decision-making processes. Specifically, CFD plays a crucial role in identifying hemodynamic alterations. CFD models were meticulously calibrated to ensure accurate representation by leveraging diverse examination data and various parameters.

CFD simulations utilize clinical imaging data, such as results of ultrasound, OCT, CT, and MRI, to derive patient-specific estimates of crucial hemodynamic parameters, including the flow rate, pressure, fractional flow reserve, and WSS (161–163). After microcirculatory disturbances in the artery, there is a decrease in blood flow and translesional pressure drop, accompanied by an increase in the fractional flow reserve without significantly affecting the non-culprit branches (161). Nonetheless, severe stenosis in the large branches results in elevated distal microcirculatory resistance, significantly increasing the flow velocity and instantaneous wave-free ratio in the cognate branches (164). Furthermore, CFD has been employed to evaluate the impact of stent implantation on peripheral flow status. The WSS and LDL filtration rates exhibit notable variations depending on stent geometry and are closely associated with the final treatment outcome and occurrence of stent restenosis, which were previously challenging to assess *in vivo* (136). In static CFD simulation, the removal of side branches with a radius of less than 50% of the parent vessel has a negligible effect on the accuracy of the fractional flow measurement. Similarly, in transient CFD simulation, the impact remains minor. This observation contributes to the simplification of CFD model construction and adjusts for the effects of geometric variations of the surrounding arteries (165). CFD techniques have been extensively applied in atherosclerosis studies owing to their practicality and effectiveness. This has led to the derivation of numerous significant parameters or metrics, enabling a more visual assessment of CVD.

The WSS is the frictional force exerted by flowing blood on the vessel wall (6). TA-WSS and wall shear stress gradient (WSSG) are vital indicators reflecting the local hemodynamic state. Compared with static simulations, TA-WSS can reflect the average changes in WSS across cardiac cycles (105). Studies have shown that regions with high TA-WSS and low oscillatory shear index have larger necrotic core sizes, larger macrophage areas, and thinner fibrous caps in atherosclerotic plaques (166). Moreover, local TA-WSS is significantly higher in patients with ischemic stroke or transient ischemic attack than in patients with asymptomatic carotid artery stenosis, indicating that TA-WSS may help stratify the risk of vulnerable carotid plaques (167).

The WSSG quantifies the magnitude of changes in the WSS along blood vessels. Under normal physiological conditions, a minimal gradient of shear stress is exerted by blood flow on the inner vessel wall (i.e., low WSSG) (168). Changes in the flow state occur when the blood vessel branches or narrows, resulting in elevated WSS and subsequent a high WSSG (169). An increase in WSSG levels is closely related to EC damage, heightened vascular permeability, and inflammatory infiltration, thus having a role in the formation and progression of atherosclerosis. In addition, WSSG is widely used in predicting arterial aneurysms. A high WSS influences the formation of MCA aneurysms, and positive WSSG promote this progression (170, 171).

Unlike the WSS, the axial plaque stress (APS) is calculated by isolating the axial component of the force exerted on the lumen or the plaque. The APS provides insights into the stress experienced by the plaque along the axis of the blood vessel and is affected by factors such as plaque shape, core stiffness, length of the lipid core, severity of the plaque, and vascular remodeling. Notably, in some studies the APS was significantly higher than the WSS (172–174). By constructing a CFD model of coronary artery blood flow, researchers discovered a linear correlation between upstream APS variation and the severity of vascular stenosis (173, 174). Alegre-Martinez et al. further elucidated that APS affects the risk of atherosclerotic plaque rupture and is related to the predilection for specific sites of plaque rupture (172). Another study reported that APS is an independent predictor of high-risk plaques and an independent risk factor for acute coronary syndrome (175).

Translesional pressure gradients are important indicators of arterial hemodynamics. Changes in blood flow in front of and behind the atherosclerotic plaques are linked to the risk of plaque rupture. Studies use the translesional pressure ratio (PR = Pressure_post-stenotic_/Pressure_pre-stenotic_) as a surrogate indicator to describe translesional pressure gradient, with PR ≤ median defined as low PR, indicating a larger translesional pressure gradient (176, 177). Through the construction of CFD models, Leng et al. found that a low PR was an independent risk factor for recurrent stroke in patients with sICAS (177). Another study showed that lower systolic blood pressure may be associated with an increased risk of stroke recurrence in patients with larger translesional pressure gradients (176). More studies are warranted to clarify the relationship between translesional pressure gradient and atherosclerosis-related diseases to guide better prevention and interventions.

In 2007, Cancel et al. demonstrated *in vitro* that under convective conditions, leaky junctions of ECs were the main pathway for LDL uptake by arteries (>90%) (178). Low WSS reduces the expression of microfilament bundles and disrupts the barrier function of ECs, causing leaky junctions between ECs and subsequently resulting in LDL infiltration into the subendothelium (11). In 2018, a study showed that the concentration of the LDL boundary layer increased when recirculation occurred near the wall and that hypertension intensified this effect by promoting the entrapment of a higher number of LDL particles (179). This may provide a foundation for LDL infiltration into the subintima of the arteries. Roustaei et al. further explained that the reason for the increased LDL infiltration rate in hypertension is the effect of reduced WSS on the number of leaky junctions and the impact of FSI on the widening of endothelial pores (180). This revealed the profound impact of hemodynamics on lipid trans-wall transport and EC function, verifying an important basis for the development of atherosclerosis (Figure 2, right panel).

Nanoparticles serve as transport media and carriers for diverse targeting substances. Researchers have used specially labeled nanoparticles to precisely track atherosclerotic lesions and increase the signal intensity of different imaging modalities (181). For example, anti-CD68 receptor-targeted Fe-doped hollow silica nanoparticles and iron oxide nanoparticles have been used to identify neovascularization and macrophages within plaques to evaluate plaque status (182, 183). Using nanoparticles as tracers, Hossain et al. analyzed blood flow and vascular deposition of circulating nanoparticles that recognize vascular cell adhesion molecule 1, E-selectin, and intercellular adhesion molecule 1 in the femoral artery of patients with peripheral artery disease (184). Furthermore, nanoparticles have the potential to provide precise treatment for atherosclerosis. Synthetic HDL-mediated targeted delivery of liver X-receptor agonists promotes cholesterol efflux from macrophages (185). In addition, CGS 25966 and CGS 27023A are N-sulfonamidoacetyl amino esters that non-selectively inhibit MMP-1, MMP-2, MMP-3, and MMP-9 by chelating zinc ions at the active site of the enzyme, rendering them potential therapeutic targets (186).

## 5. New biomarkers

### 5.1. Biochemical biomarkers

Abnormalities in inflammation and lipid metabolism predict the risk of atherosclerosis. Plaque inflammation and its related metabolic markers are valuable indicators of plaque stability.

Growth differentiation factor 15 (GDF-15) is a member of the transforming growth factor β superfamily, which is closely associated with lipid metabolism and inflammation. During the initial phase of atherosclerosis, researchers have observed smaller atherosclerotic lesions in GDF-15^−/−^ mice; however, this difference disappeared after 12 weeks (187). In a recent cross-sectional study, Shima et al. discovered that high-sensitivity C-reactive protein and GDF-15 levels were significantly higher in patients with coronary artery disease (*p* = 0.091 and *p* < 0.001, respectively) (188). Heduschke et al. pointed out that recombinant GDF-15 promotes macrophage autophagy activity and GDF-15^−/−^ mice show reduced macrophage autophagy activity in plaques, which may be related to plaque regression and stability (189). Ackermann et al. further demonstrated that silencing GDF-15 in human macrophages inhibits oxidized low-density lipoprotein-induced lipid accumulation (190). Overall, the role of GDF-15 in the influence of inflammation on atherosclerosis appears to be multifaceted, suggesting that GDF-15 may serve as a predictor of plaque stability (191).

Neopterin, an oxidation product of 7,8-dihydroneopterin, is produced by activated macrophages stimulated by interferon-γ released from T lymphocytes (192, 193). Co-cultivation of ox-LDL with macrophages promotes the conversion of 7,8-dihydroneopterin to neopterin, highlighting its involvement in facilitating the clearance of ox-LDL (193). Shirai et al. showed that neopterin inhibits foam cell formation, migration, and proliferation of VSMCs. Moreover, neopterin suppresses the phosphorylation of nuclear factor kappa-B transcription factor in human arterial macrophages and increases the expression of peroxisome proliferator-activated receptor γ (194). Clinical studies have shown that patients with coronary heart disease have significantly higher serum neopterin levels. In one study neopterin concentration was positively correlated with the degree of coronary artery stenosis (195). Sugioka et al. also observed that neopterin levels were significantly higher in patients with complex carotid atherosclerotic plaques than in those with non-complex plaques (196). The observed elevation in neopterin protein expression within atherosclerotic plaques is likely attributable to the endogenous upregulation of neopterin proteins, which serve as a defensive response against the progression of atherosclerosis (194).

Other novel biological markers, such as galectin-3, may serve as surrogates to reflect plaque inflammation and calcification (197, 198), additionally pregnancy-associated plasma protein-A has been associated with vulnerable plaque features in patients with coronary artery disease (199), and the count of CD16^+^ monocytes has shown a correlation with preclinical CVD (200).

### 5.2. Genetic biomarkers

Atherosclerosis is a multifactorial disease influenced by genetic, environmental, and pathophysiological factors. Multiple genes and ncRNAs regulate lipid metabolism, inflammation, and endothelial and smooth muscle cell function. Gene expression analysis can reveal the dynamic state of a disease and provide insight into its potential causes. Meng et al. compared gene expression differences between healthy individuals and patients with atherosclerosis and found that TPM2 was significantly downregulated in atherosclerotic samples (201). In another study, ITGAX, CCR1, IL1RN, CXCL10, CD163, and MMP-9 were found to be significantly upregulated in atherosclerotic samples (202). Several genes participate in the various processes associated with atherosclerosis. For instance, SVEP1 induces the proliferation of vascular smooth muscle cells (VSMCs), elevates integrin levels, and triggers plaque inflammation, thereby contributing to atherosclerosis development irrespective of blood lipid levels (203). In contrast, JCAD governs the Hippo/YAP/TAZ pathway and mediates endothelial dysfunction, thus fostering atherosclerosis (204). These genes have the potential to serve as valuable risk indicators and treatment targets in individuals with atherosclerosis.

miRNAs are a class of endogenous small single-stranded non-coding RNAs approximately 22 nucleotides in length that regulate post-transcriptional gene expression by degrading target mRNAs or blocking their translation (205). miRNAs play important roles in regulating pathological processes such as cell adhesion, proliferation, lipid uptake, efflux, and the production of inflammatory mediators (206). The upregulation of miR-9 suppresses the formation of vulnerable atherosclerotic plaques through negative regulation of the p38MAPK pathway via OLR1 and enhances vascular remodeling in mice with acute coronary syndrome (207). Moreover, miR-9 has similar functions of inhibiting SDC2 and the FAK/ERK signaling pathway, reducing the plaque area in aortic atherosclerosis (208). Furthermore, the upregulation of miR-9 decreased the levels of tumor necrosis factor-α, IL-6, and IL-1β (207, 208). MiR-181a-5p and miR-181a-3p collectively reduce the expression of pro-inflammatory cytokines, decrease macrophage, leukocyte, and lymphocyte infiltration, and block nuclear factor kappa B activation and vascular inflammation by targeting TAB2 and NEMO (209). In contrast, overexpression of miRNA-155 promotes the activation of the nucleotide-binding oligomerization domain-like receptor protein 3 inflammasome induced by ox-LDL, exacerbating atherosclerosis in ApoE^−/−^ mice (210). Clinical studies have shown that compared with carotid atherosclerosis patients with stable plaques, miR-223 and miR-126 are downregulated in patients with unstable plaques (211). Besides, MiR-21 is highly positively correlated with the maximum lipid core area, number of lesion vessels, number of macrophages, and number of vulnerable plaques in patients with acute coronary syndrome and negatively correlated with fibrous cap thickness (212). These findings indicate the potential use of miRNAs as predictive indicators of plaque stability.

In addition to miRNAs, other non-coding RNAs, such as long non-coding RNA and circular RNA, have garnered growing attention owing to their involvement in atherosclerosis (205). Furthermore, single mutations, small insertions/deletions, and copy number variants in genes related to lipid metabolism are also associated with atherosclerosis (213). The regulation of genes related to lipid metabolism, vascular inflammation, and EC function is influenced not only by genetic factors, but also by changes in DNA methylation caused by environmental factors. These epigenetic changes may contribute to atherosclerosis (214).

## 6. Conclusion and prospect

The high costs of advanced imaging technologies such as PET, IVUS, NIRF, and NIRS are significant factors that limit their widespread use, and it is expected that this problem will be addressed in the future. Further exploration is needed to improve the accuracy and resolution of noninvasive examinations. Currently, various examination techniques capture the distinct characteristics of plaques and blood vessels, creating a vibrant area of research focused on integrating these techniques to enhance the understanding of plaques and blood vessels. The combination of emerging digital simulation and imaging techniques presents exciting opportunities in the field of atherosclerosis research. Consequently, the adoption of new patient-specific evaluation of hemodynamic or biomechanical parameters offers a fresh perspective for assessing CVD risk.

In conclusion, atherosclerotic plaques are an important cause of CVD. The location, growth, and properties of plaques are critical factors influencing clinical outcomes. In clinical practice, technological advancements have provided valuable insights into plaque composition, and the development of quantitative and qualitative analytical techniques holds promise for a more efficient and precise understanding of atherosclerosis. Accurate and scientific prediction of plaque rupture risk necessitates the integration of multiple indicators, including quantitative scoring of plaques and hemodynamic evaluation of arteries. The implementation of preventive measures and timely intervention can mitigate the impact of cardiovascular disease on patients’ lives. Analysis of plaque formation and progression may offer valuable insights and potential strategies for diagnosis and prevention.

## Author contributions

ZH and JL drafted the original manuscript. QL, ML, and WK participated in the revision of the review. XN and ZZ reviewed and edited the review. All authors contributed to the article and approved the submitted version.

## Figures and Tables

**Table:** 

CVD	cerebrovascular or cardiovascular diseases
LDL	low-density lipoprotein
IMT	intima-media thickness
EC	endothelial cell
VSMCs	vascular smooth muscle cells
WSS	wall shear stress
IPH	intraplaque hemorrhage
BA	basilar artery
TPA	total plaque area
TPV	total plaque volume
CT	computed tomography
MRI	magnetic resonance imaging
IVUS	intravascular ultrasound
OCT	optical coherence tomography
PWI	pulse wave imaging
PET	positron emission tomography
NIRS	near infrared spectroscopy
IL	interleukin
MMP	matrix metalloproteinase
ox-LDL	oxidized low-density lipoprotein
ISD	inter-slice distance
CD	cluster of differentiation
GDF-15	growth differentiation factor 15
CFD	computational fluid dynamics
CAC	coronary artery calcium
ICAS	intracranial atherosclerosis
HR-MRI	high-resolution MRI
MCA	middle cerebral artery
NIRF	near-infrared fluorescence
FSI	fluid–structure interaction
WSSG	wall shear stress gradient
TA-WSS	time-averaged WSS
APS	the axial plaque stress
PR	pressure ratio
